# Magnetic and Structural Properties of Barium Hexaferrite BaFe_12_O_19_ from Various Growth Techniques

**DOI:** 10.3390/ma10060578

**Published:** 2017-05-25

**Authors:** Denis A. Vinnik, Aleksandra Yu. Tarasova, Dmitry A. Zherebtsov, Svetlana A. Gudkova, Damir M. Galimov, Vladimir E. Zhivulin, Evgeny A. Trofimov, Sandra Nemrava, Nikolai S. Perov, Ludmila I. Isaenko, Rainer Niewa

**Affiliations:** 1 Laboratory of Single Crystal Growth, South Ural State University, Chelyabinsk 454080, Russia; vinnikda@susu.ru (D.A.V.); aleksandra_tarasova@mail.ru (A.Y.T.); zherebtcovda@susu.ac.ru (D.A.Z.); svetlanagudkova@yandex.ru (S.A.G.); galimovdm@susu.ac.ru (D.M.G.); zhivulinve@mail.ru (V.E.Z.); trofimovea@susu.ac.ru (E.A.T.); lisa@igm.nsc.ru (L.I.I.); 2 Institute of Geology and Mineralogy, Siberian Branch Russian Academy of Sciences, Novosibirsk 630090, Russia; 3 SEC Nanotechnology, Moscow Institute of Physics and Technology (State University), Dolgoprudny, Moscow Region 141701, Russia; 4 Physics Department, Chelyabinsk State Pedagogical University, Chelyabinsk 454080, Russia; 5 Institute of Inorganic Chemistry, University of Stuttgart, Stuttgart 70569, Germany; sandra.nemrava@iac.uni-stuttgart.de; 6 Faculty of Physics, Moscow State University, Moscow 119991, Russia; perov@magn.ru; 7 Laboratory of Semiconductor and Dielectric Materials, Novosibirsk State University, Novosibirsk 630090, Russia

**Keywords:** inorganic compounds, magnetic materials, crystal growth, crystal structure, magnetic properties

## Abstract

Barium hexaferrite powder samples with grains in the μm-range were obtained from solid-state sintering, and crystals with sizes up to 5 mm grown from PbO, Na_2_CO_3_, and BaB_2_O_4_ fluxes, respectively. Carbonate and borate fluxes provide the largest and structurally best crystals at significantly lower growth temperatures of 1533 K compared to flux-free synthesis (1623 K). The maximum synthesis temperature can be further reduced by the application of PbO-containing fluxes (down to 1223 K upon use of 80 at % PbO), however, Pb-substituted crystals Ba_1–*x*_Pb*_x_*Fe_12_O_19_ with Pb contents in the range of 0.23(2) ≤ *x* ≤ 0.80(2) form, depending on growth temperature and flux PbO content. The degree of Pb-substitution has only a minor influence on unit cell and magnetic parameters, although the values for Curie temperature, saturation magnetization, as well as the coercivity of these samples are significantly reduced in comparison with those from samples obtained from the other fluxes. Due to the lowest level of impurities, the samples from carbonate flux show superior quality compared to materials obtained using other methods.

## 1. Introduction

The increase in electromagnetic pollution due to the rapid development of gigahertz (GHz) electronic systems and telecommunication has resulted in a growing and intense interest in electromagnetic absorber technology. Electromagnetic interference (EMI) can cause severe interruption of electronically controlled systems. It can produce device malfunctions, generate false images, increase clutter on radar, and reduce performance because of system-to-system coupling. These are some of the reasons why the use of self-generated electromagnetic radiation apparatuses, which include cellular telephones and wireless computers, are strictly prohibited in certain areas, for example, in hospitals, banks, petrol stations and inside airplanes. To overcome the problems created by EMI, electromagnetic wave absorbers with the capability of absorbing unwanted-undesirable electromagnetic signals are used, and research on their electromagnetic and absorption properties is in progress [1,2]. Recent developments in microwave absorber technology have resulted in materials with high wave absorption coefficients, good physical performance, and lower production costs [3,4]. As far as thickness and working frequency bandwidth are concerned, magnetic composites have obvious advantages. The magnetic fillers often used in such composites are ferrite materials, such as spinel ferrites and hexaferrites [5,6].

Hexaferrites with planar magnetic anisotropy are widely used as electromagnetic wave absorbers in the GHz range. Barium hexaferrite powders are ideally suited fillers for the development of electromagnetic attenuation materials at microwave frequencies, due to their low cost, low density, high stability, large electrical resistivity, and high microwave magnetic loss. Thus, they find applications as electromagnetic emission absorber materials [7,8,9,10,11,12,13,14,15].

It is well known that the magnetic properties and, in particular, the anisotropy field *H*_а_ of M-type (magnetoplumbite crystal structure) Ba hexaferrite can be changed by substituting Fe^3+^ ions, resulting in a shift in resonance frequency [1,16,17]. This fact led to large efforts to modify the magnetic parameters of barium hexaferrite by substitution with other cations or cation combinations, either exclusively on the iron site, or simultaneously on both the iron and the barium sites. In all such modified ferrites, it is necessary that substituted ions maintain electrical neutrality and also have similar ionic radii to the original one [17]. A wide range of possible compositions of these ferrites were synthesized by various synthesis techniques [18,19,20]. Still, little is known about the influence of substitution by nonmagnetic ions on the Ba site or by impurities such as sodium. However, these influences as well as grain size and crystal quality are known to have a significant impact on the magnetic parameters.

In recent years, the technology of barium hexaferrite synthesis via various techniques has greatly improved, but the main obstacle in crystal growth continues to be the high melting temperature of BaFe_12_O_19_ of 1580 ± 50 °С [21]. Current developments include the synthesis of barium and strontium hexaferrites in carbon monoxide reductive gas followed by recalcination [22], or from glass crystallization using a mixture of antimony and boron oxides [23]. An important task is to find a solvent or flux capable of both lowering the melting point, and ensuring the crystallization of barium hexaferrite phases. Of particular interest are carbonate fluxes [24,25], borate fluxes [26,27], and lead oxide fluxes [26,28], which led to promising results. However, little detailed data about PbO solvent use has been presented. We studied the effect of different concentrations of lead oxide on the homogenization temperature of the solution, as well as the composition, structure, and properties of the resulting barium hexaferrite crystals. Furthermore, we compared the results to those from crystals obtained from carbonate and borate fluxes as well as from solid-state sintering without the presence of a flux. In this respect, it may not be surprising that PbO flux leads to the partial substitution of Ba^2+^ by Pb^2+^, since the eponym of the material with a magnetoplumbite-type crystal structure is the Pb-containing mineral PbFe_12_O_19_ [29].

## 2. Materials and Methods 

### 2.1. Synthesis and Crystal Growth

Binary iron and lead oxide, barium oxoborate, as well as barium and sodium carbonate were used as initial components for single crystal growth. For the initial compositions of the different experiments, see Table 1. After grinding the mixtures together in an agate mortar, they were placed in a 30-mL platinum crucible within a resistive furnace. A detailed description of the furnace was published earlier [30]. In case of flux growth, the furnace was maintained at 1533 K for 3 h followed by cooling at a rate of 4.5 K/h to 1173 K to homogenize the starting materials. The system was then allowed to naturally cool to room temperature. The spontaneously obtained crystals with sizes of up to 7 mm were separated from the solidified melt by leaching in hot nitric acid to remove side phases. For solid-state sintering, mixtures of Fe_2_O_3_ and BaCO_3_ were heated to 1623 K and held for 3 h.

### 2.2. Characterization

To investigate the composition, structure, and properties of the samples, they were examined by the following methods: The chemical compositions of the grown samples were determined with use of an electron scanning microscope with an energy dispersive spectrometer for elemental analysis (Jeol JSM7001F/Oxford INCA X-max 80, Tokyo, Japan). X-ray powder diffraction analysis was carried out with filtered Cu *Kα* radiation (RigakuUltima IV, Tokyo, Japan). To determine the crystallographic details of the Pb-substituted samples, single crystal X-ray diffraction intensities were collected with monochromatic Mo *Kα* radiation (NONIUS κ-CCD). This information is presented in the supplementary material. Magnetic properties were examined on a differential scanning calorimeter for determination of *T*_C_ (Netzsch 449C Jupiter, Netzsch, Selb, Germany) and a vibrating sample magnetometer (VSM LakeShore 7407, Lake Shore Cryotronics Inc., Westerville, OH, USA).

## 3. Results

### Solid-State Sintering

Barium hexaferrite crystals were grown in the absence of a flux as well as from Na_2_CO_3_, BaB_2_O_4_, and PbO fluxes to analyze the crystal quality, size, and the resulting magnetic properties. In the case of the PbO flux, a significant substitution of Ba by Pb depending on the growth temperature and flux concentration was observed. Table 1 presents the initial charge compositions, general compositions according to single crystal X-ray diffraction structure refinements, as well as the maximum temperature of the crystal growth process. The obtained chemical compositions were additionally validated using energy dispersive X-ray spectroscopy (EDX) analysis: For all crystals, the derived Fe/(Ba + Pb) ratio was equal to 12 ± 0.2. For the crystals obtained from PbO flux, the Pb content resulted in *x* = 0.21, 0.45, and 0.81 for a general formula Ba_1–*x*_Pb*_x_*Fe_12_O_19_, compared to 0.23, 0.44, and 0.80, respectively, from refinements of X-ray diffraction data obtained on single crystals.

## 4. Discussion

### 4.1. Solid-State Sintering

**Figure 1:**
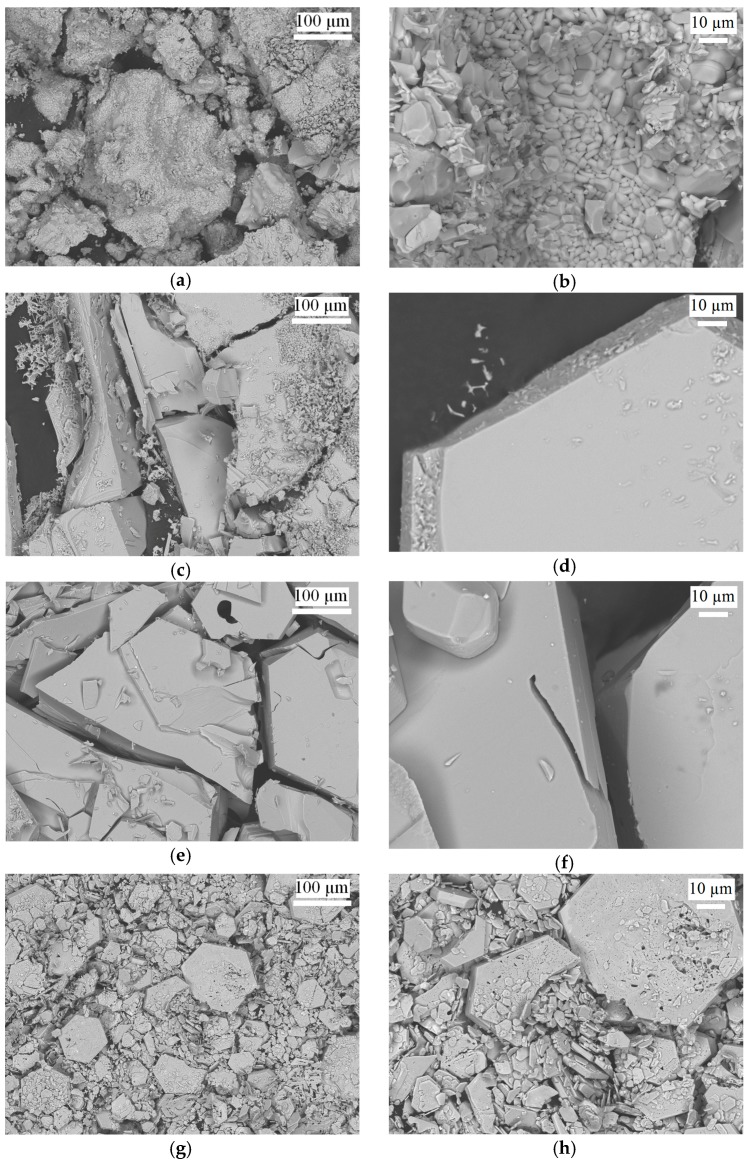
SEM images of barium hexaferrite samples from: (**a**,**b**) solid-state sintering; (**c**,**d**) Na_2_CO_3_ flux; (**e**,**f**) BaB_2_O_4_ flux; (**g**,**h**) 60 at % PbO flux (see Table 1).

**Figure 2:**
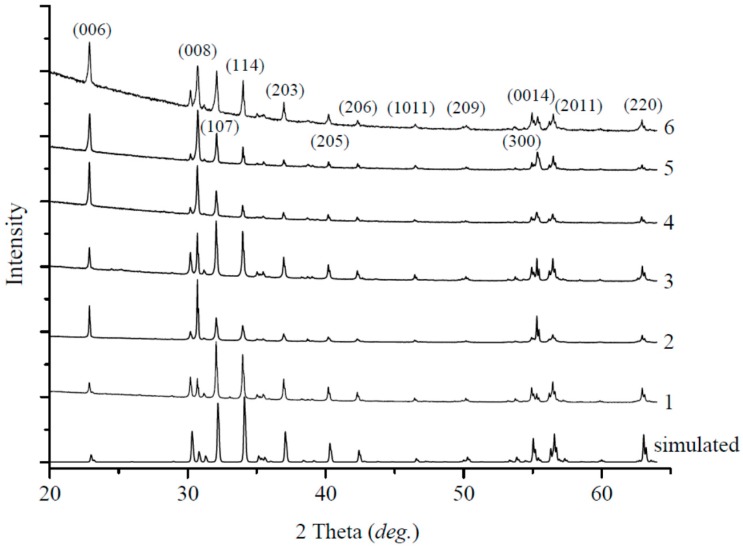
Powder XRD patterns of experimental samples and literature data (bottom) [32]. Differences arise due to minor variations in intensities according to the substitution of Ba by Pb, and mostly different degrees in preferred orientation of grains.

The optimal temperature of the solid-state synthesis of Ba hexaferrite, which ensures a stable formation and crystal growth, was earlier established experimentally to be 1623 K [31]. Figure 1 shows the crystals obtained at this temperature from mixtures of barium carbonate and iron(III) oxide to range within the μm-size. Within this study, these are the smallest crystallites. While the highest crystal growth temperature was necessary, this underlining the effect of higher diffusion at already lower temperatures in a flux. Powder X-ray diffraction (PXRD) patterns proof the purity of the product. Unit cell parameters are virtually identical to those reported in the literature (Table 2). Figure 2 presents the powder XRD patterns of the experimental samples and the literature data [32].

### 4.2. Carbonate and Borate Flux Growth 

Crystal growth experiments from Na_2_CO_3_ and BaB_2_O_4_ fluxes were carried out at 1533 K, thus 90 K below the solid-state sintering experiment. Under these conditions, Na_2_CO_3_ eventually decomposes to form Na_2_O. Still, crystal sizes and visual quality were significantly improved (see Figure 1, second and third row).

The most complete analysis of parameters in the BaO-Fe_2_O_3_-Na_2_O system, ensuring the growth of barium hexaferrite, was carried out in 1961 by R. Gambino and F. Leonhard [24]. The optimal ratio of "crystal/solvent" was derived to 73.7/26.3 at %, very close to our initial ratio in the crystal growth experiment. SEM images (Figure 1) indicate a good surface morphology of the obtained crystals easily approaching the mm-size range, which represent the largest crystals obtained within this study.

The general possibility of barium hexaferrite crystal growth using BaB_2_O_4_ as a flux has been established more recently [27]. Experimentally, it was found that a mixture with 30 at % BaB_2_O_4_ provides a homogeneous melt at 1533 K. The obtained crystals are only slightly smaller than those from carbonate flux and exhibit a similarly good surface morphology with an apparent low surface defect density.

### 4.3. PbO Flux Growth 

**Figure 3:**
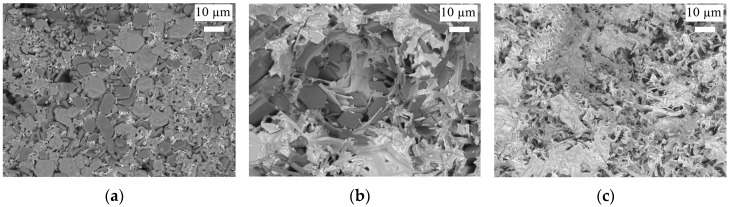
SEM images of barium hexaferrite single crystals grown from various PbO fluxes: (**a**) PbO 60 at %, 1473 K; (**b**) PbO 70 at %, 1298 K; (**c**) PbO 80 at %, 1223 K.

Barium hexaferrite crystals previously have been grown from PbO flux at 1473 K [26]. To investigate the optimal temperature for the growth process applying PbO flux, experiments were carried out with different flux contents of 60, 70, and 80 at % PbO. The mixtures were apparently molten at 1473, 1298, and 1223 K, respectively, and then rapidly cooled to room temperature, during which crystallization occurred. In all experiments, faceted hexagonal crystals of several μm were obtained, however, the amount of crystals and their size decreased with increasing PbO concentration (Figure 3). According to PXRD analysis, the samples are single phase and of magnetoplumbite-type structure (Figure 2).

As was earlier observed [26], the PbO flux causes the growth of crystals with partial substitution of Ba by Pb, resulting in a general composition of Ba_1–*x*_Pb*_x_*Fe_12_O_19_. Unit cell determination from powder diffraction (Table 2) already indicates a composition dependence of the lead content *x* on the flux composition and growth temperature. Due to the larger ionic radius of Ba^2+^ compared with Pb^2+^ (*r*(Ba^2+^) = 1.61 Å; *r*(Pb^2+^) = 1.49 Å with CN = 12 [37]), a decreasing unit cell with increasing *x* is expected. However, upon lower PbO concentrations in the flux (60 at %) the unit cell is even very slightly enlarged, but almost does not change with increasing Pb content. Due to the rigid iron oxide framework with larger Ba^2+^ ions as spacers, the unit cell generally hardly changes upon partial substitution by Pb^2+^. However, the pure lead compound has a significant about 1.5% smaller unit cell [36]. In order to obtain a more reliable Pb content incorporated in the crystals, we have undertaken full single crystal structure determinations (see below). One result is the increasing Pb content with increasing Pb concentration in the flux from Ba_0.77(2)_Pb_0.23_Fe_12_O_19_ at 60 at % PbO over Ba_0.56(2)_Pb_0.44_Fe_12_O_19_ at 70 at % PbO to Ba_0.20(3)_Pb_0.80_Fe_12_O_19_ at 80 at % PbO. Earlier, an approximate composition of Ba_0.9_Pb_0.1_Fe_12_O_19_ was discussed for a PbO flux proportion of 50 at % at 1473 K [26].

**Figure 4:**
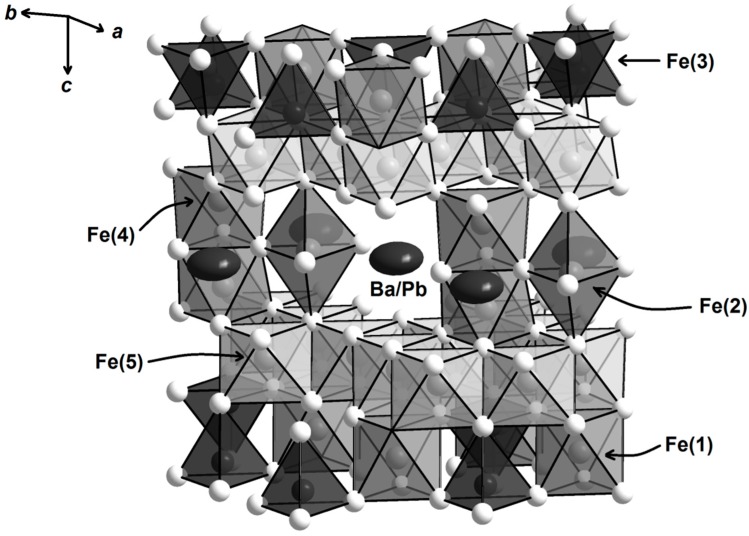
Section of the crystal structure of Ba_1–x_Pb_x_Fe_12_O_19_.

**Figure 5:**
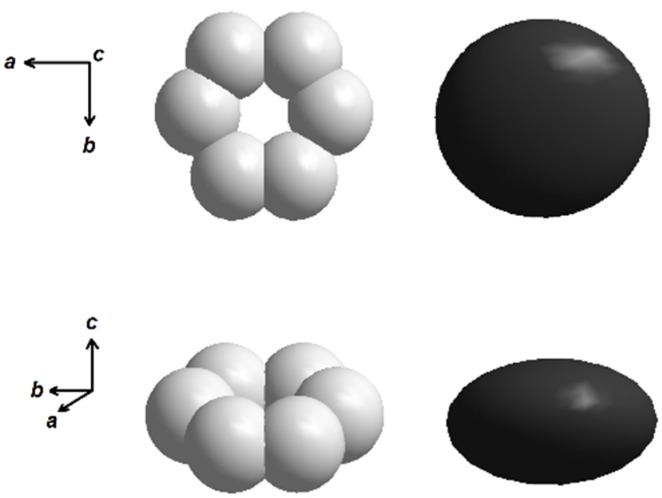
Flattened displacement parameter for the mixed occupied site Ba/Pb in the crystal structure of Ba_1–x_Pb_x_Fe_12_O_19_ (**right**), and partially occupied six-fold split-positions for Pb (**left**) [36].

Single crystal X-ray diffraction data result in compositions Ba_1–*x*_Pb*_x_*Fe_12_O_19_ with no indication for any Ba/Pb order. Close inspection of the refinement results revealed a large flat displacement ellipsoid for the mixed occupied Ba/Pb site (Figure 4). Such enlarged displacement parameters for Pb were earlier observed for pure PbFe_12_O_19_ [36]. With respect to an apparently stereochemically active electron lone pair of Pb^2+^, a displacement of this ion from the ideal 2*d* site (invariant 2/3, 1/3, 1/4) to the 12*j* site (*x*, *y*, 1/4) within the space group *P*6_3_/*mmc* is discussed, at which Pb is equally distributed over six split-positions resulting in an occupancy of 1/6 (Figure 5). Due to the partial occupation of the central ideal 2*d* site by Ba, these split-positions are not sufficiently separated to resolve them from the ideal position and refine them independently. The split-positions are located within the hexagonal *ab*-plane and thus lead to the observed enlargement of the displacement ellipsoid with the observed shape if modeled with one site at the ideal position for Ba and Pb. Further information on the crystal structure refinements can be found in the supplementary material.

### 4.4. Magnetic Properties

**Figure 6:**
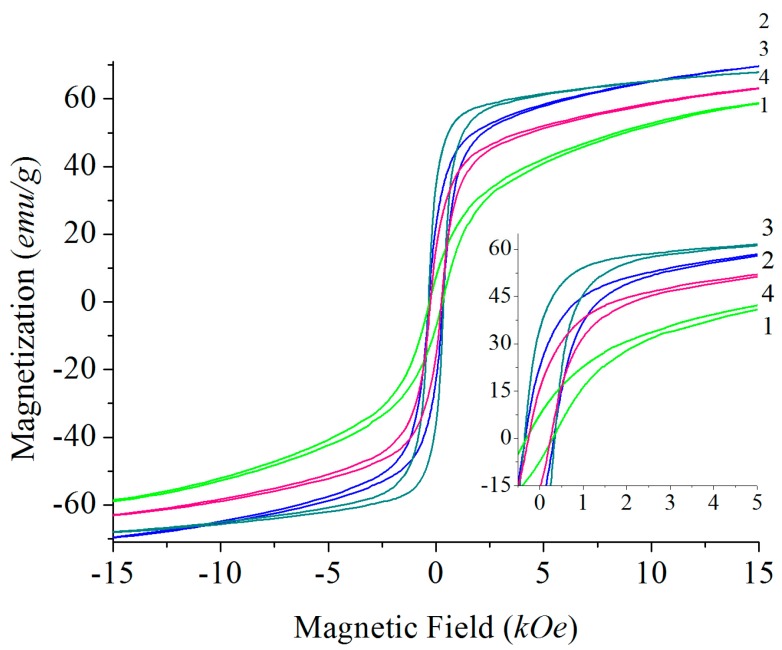
Hysteresis loops of barium hexaferrite samples BaFe_12_O_19_: 1 (light green), obtained from solid-state sintering; 2 (blue), from carbonate flux; 3 (dark green), from borate flux; and 4 (red), from PbO flux (composition Ba_0.77(2)_Pb_0.23_Fe_12_O_19_, compare with Table 2). Insert figure: Detailed view of the section between 0 and 5 kOe.

**Figure 7:**
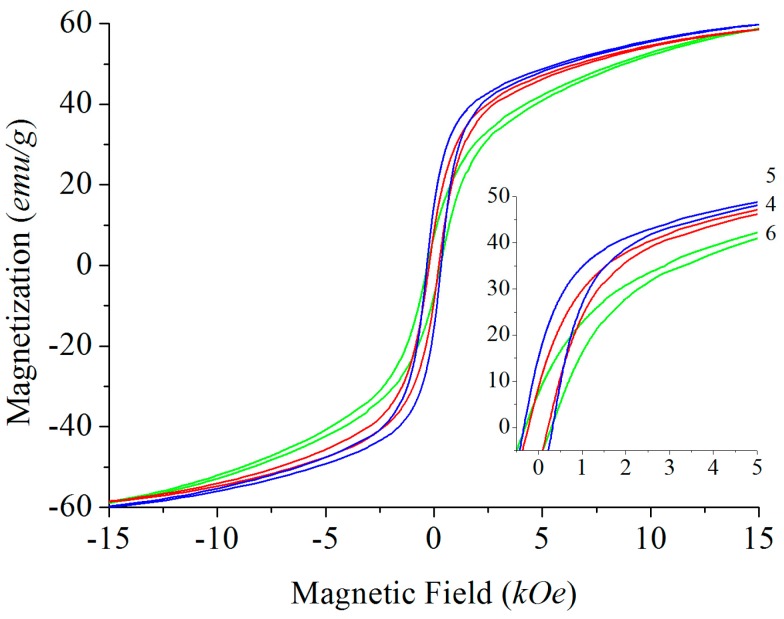
Hysteresis loops of Pb-substituted barium hexaferrite samples Ba_1–*x*_Pb_x_Fe_12_O_19_ with *x* = 0.23(2), 0.44(2), and 0.80(2) for 4 (red), 5 (blue), and 6 (green), respectively (compare with Table 2). Insert figure: Detailed view of the section between 0 and 5 kOe.

Magnetic hysteresis loops taken at room temperature for all discussed samples are presented in Figure 6 and Figure 7. Important magnetic parameters such as Curie temperature (derived from DSC (differential scanning calorimetry) measurements), saturation magnetization, and coercivity are also summarized in Table 2. Literature data on magnetic characteristics of BaFe_12_O_19_ vary within comparatively large ranges [34−36] and apparently depend on the applied synthesis technique, however, they are consistent with our data. As observed earlier, the saturation magnetization of flux-grown samples is higher than those of powder-sintered materials [38]. The coercivity of powder-sintered samples differs significantly from those grown from flux, although the *M*_s_ values are very close. This observation can be explained since the coercivity depends on the grain sizes (Figure 1).

The samples grown from Na_2_CO_3_ (Na_2_O, respectively) and BaB_2_O_4_ fluxes show the highest values of *H*_c_, which are in agreement with earlier derived numbers for materials obtained from such fluxes [35] and are likely due to the low impurity levels. As a result of partial substitution of Ba by Pb, all samples grown from PbO flux exhibit reduced values of all magnetic properties. However, there is hardly any significant dependence of magnetic properties on the Pb content achieved in our study (Figure 7).

## 5. Conclusions

Barium hexaferrite samples BaFe_12_O_19_ with sizes up to 5 mm grown from PbO, Na_2_CO_3_, and BaB_2_O_4_ fluxes as well as μm-size powder-sintered samples were investigated. Fluxes are able to reduce the growth temperature compared to solid-state sintering, but may supply impurities or additional challenges due to evaporation. The Na_2_CO_3_ flux method provides the largest crystals and can be used to obtain materials of superior quality, since coercivity and magnetization suffer from a strong dependence on impurity values. This study shows the possibility to control the magnetic properties of BaFe_12_O_19_ in a wide range by using different synthesis techniques.

For PbO-containing fluxes, the lowest melting temperature of 1223 K was observed for a solution with 80 at % PbO, which is significantly lower than the melting temperature of pure barium hexaferrite (1853 K) [21]. However, the Pb content increases with flux Pb content. According to magnetization measurements, the substitution by Pb reduces the values for Curie temperature, saturation magnetization, and coercivity, but these important material characteristics do not significantly depend on the degree of substitution.

From the materials grown in this study, the largest single crystals obtained from Na_2_O flux are optimal for the production of spherical isolator elements. The crystals showed diameters up to 7 mm and thicknesses up to 5 mm. BaB_2_O_4_ and PbO fluxes can be used for the production of single crystals with large aspect ratios for flat/plane attenuator manufacturing, since crystal diameters ranged up to 5 mm, while typically exhibiting thicknesses below 1 mm. However, lead oxide shows a high evaporation rate at the applied growth temperatures, and thus presents environmental issues during its use as a flux as well as in the final material lead content. The solid-state sintering synthesis is ideally suited for the cost-effective production of powder for absorbing coatings.

## Figures and Tables

**Table 1:** Initial molar ratios of charge compositions (at %) and maximum temperatures of the different crystal growth experiments. Resulting compositions originate from full structure refinements based on single crystal X-ray diffraction intensity data.

No.	BaCO_3_	Fe_2_O_3_	Flux	Composition	*T*, °С
1	14.3	85.7	-	BaFe_12_O_19_	1350
2	10.5	63.2	26.3 Na_2_CO_3_	BaFe_12_O_19_	1260
3	60.0	10.0	30.0 BaB_2_O_4_	BaFe_12_O_19_	1260
4	5.7	34.3	60.0 PbO	Ba_0.77(2)_Pb_0.23_Fe_12_O_19_	1200
5	4.3	25.7	70.0 PbO	Ba_0.56(2)_Pb_0.44_Fe_12_O_19_	1025
6	2.9	17.1	80.0 PbO	Ba_0.20(3)_Pb_0.80_Fe_12_O_19_	950

**Table 2:** Unit cell parameters from PXRD, Curie temperature, and saturation magnetization values of powder samples of barium hexaferrite.

No.	Synthesis Method	Pb Content *x*	*a* (Å)	*c* (Å)	*V* (Å³)	*T*_C_ (K)	*M*_s_ (emu/g)	*H*_c_ (Oe)
[32]			5.893	23.194	697.5	-	-	-
[33]			-	-	-	730	-	-
[34]			-	-	-	-	72.0	5395
[35]			-	-	-	-	59.0	360
1	Solid-state	0	5.8922(1)	23.1953(6)	697.40(2)	726	63.5	254
2	Na_2_CO_3_ flux	0	5.8929(4)	23.194(2)	697.54(6)	728	71.0	363
3	BaB_2_O_4_ flux	0	5.8915(2)	23.1917(8)	697.13(4)	725	68.0	348
4	60 at % PbO flux	0.23(2)	5.8962(4)	23.1927(1)	698.28(6)	721	59.3	299
5	70 at % PbO flux	0.44(2)	5.8948(3)	23.1780(8)	697.51(4)	722	60.1	328
6	80 at % PbO flux	0.80(2)	5.8917(9)	23.173(3)	696.60(19)	724	58.8	223
[36]	PbFe_12_O_19_	1	5.873	23.007	687.24			

## Supplementary Materials

The following are available online at www.mdpi.com/1996-1944/10/6/578/s1. Tables S1−S7: Crystal structure data for Pb-substituted barium hexaferrites Ba_0.20(2)_Pb_0.80_Fe_12_O_19_, Ba_0.56(2)_Pb_0.44_Fe_12_O_19_, and Ba_0.77(2)_Pb_0.23_Fe_12_O_19_.
