# Supplementary material for: Magnetic and Structural Properties of Barium Hexaferrite BaFe12O19 from Various Growth Techniques
Source: Materials (Basel). 2017 May 25;10(6):578. doi: 10.3390/ma10060578 (PMC5552085; doi:10.3390/ma10060578)
Supplement: Supplementary file 1 [file materials-10-00578-s001.pdf]

Supplementary material

Table S1. Measured and calculated data of the structure Pb-substituted barium hexaferrite

	$Ba_{0.20(3)}Pb_{0.80}Fe_{12}O_{19}$	$Ba_{0.56(2)}Pb_{0.44}Fe_{12}O_{19}$	$Ba_{0.77(2)}Pb_{0.23}Fe_{12}O_{19}$
Symmetry	hexagonal	hexagonal	hexagonal
Space group	$P6_3/mmc$	$P6_3/mmc$	$P6_3/mmc$
a [Å]	5.8942(2)	5.8902(2)	5.8947(1)
c [Å]	23.1591(7)	23.1692(10)	23.1985(7)
Z	2	2	2
$Density_{calc}$ [g/cm³]	5.562	5.449	5.449
Volume [Å³]	696.79(4)	696.15(4)	698.09(3)
Diffractometer	NONIUS κ-CCD	NONIUS κ-CCD	NONIUS κ-CCD
Radiation [Å]	$MoK_{\alpha 1}$; λ = 0.7107	$MoK_{\alpha 1}$; λ = 0.7107	$MoK_{\alpha 1}$; λ = 0.7107
Range *hkl*	± 7, ± 7, ± 30	± 7, ± 7, -29 < *l* < 28	- 7 < *h* < 6 , ± 7, ± 30
Θ_{max} [deg]	55.74	53.99	55.67
F(000)	1081.6	1062.9	1052.0
$\mu(MoK_\alpha)$ [mm^{-1}]	22.37	19.06	17.07
Reflections measured/independent	13064/373	12217/340	13312/374
R_{int}/R_σ	0.0730/0.0194	0.0792/0.0237	0.0722/0.0186
$R_1 \| F_o \geq 4\sigma(F_o)$	0.0607	0.0543	0.0434
R_1/wR_2/GooF	0.0538/0.1164/1.166	0.0413/0.1104/1.121	0.0339/0.0806/1.139
Max. and min. residual electron density [Å^{-3}]	4.68 and – 2.24	2.39 and – 0.94	2.29 and – 0.94

Table S2. Atomic coordinates, occupation factors and isotropic displacement parameters (pm²) for $Ba_{0.20(3)}Pb_{0.80}Fe_{12}O_{19}$

Atom	Site	*x*/*a*	*y*/*b*	*z*/*c*	Occ. factor	U_{iso}
Ba/Pb	2*d*	2/3	1/3	¼	0.20(3)/0.80	539(10)
Fe(1)	2*a*	0	0	0	1	63(9)
Fe(2)	2*b*	0	0	1/4	1	153(11)
Fe(3)	4*f*	2/3	1/3	0.5273(1)	1	61(7)
Fe(4)	4*f*	2/3	1/3	0.6898(1)	1	69(7)
Fe(5)	12*k*	0.1688(2)	2*x*	0.6084(1)	1	60(5)
O(1)	4*e*	0	0	0.6504(6)	1	40(27)
O(2)	4*f*	2/3	1/3	0.4453(6)	1	55(29)
O(3)	6*h*	0.1826(16)	2*x*	¼	1	174(29)
O(4)	12*k*	0.1570(9)	2*x*	0.0523(3)	1	61(16)
O(5)	12*k*	0.5027(10)	2*x*	0.1496(3)	1	79(16)

Table S3. Anisotropic thermal parameters (pm²) for $Ba_{0.2(2)}Pb_{0.8}Fe_{12}O_{19}$

Atom	U_{11}	U_{22}	U_{33}	U_{23}	U_{13}	U_{12}	U_{iso}
Ba/Pb	687(14)	U_{11}	247(10)	0	0	343(7)	539(10)
Fe(1)	61(13)	U_{11}	68(18)	0	0	31(6)	63(8)
Fe(2)	155(16)	U_{11}	148(21)	0	0	78(8)	153(11)
Fe(3)	82(8)	U_{11}	85(11)	0	0	31(5)	62(7)
Fe(4)	58(10)	U_{11}	91(13)	0	0	29(5)	69(7)
Fe(5)	49(7)	39(9)	88(8)	-1(1)	½ U_{23}	20(4)	60(5)

Table S4. Atomic coordinates, occupation factors and isotropic displacement parameters (pm²) for $Ba_{0.56(2)}Pb_{0.44}Fe_{12}O_{19}$

Atom	Site	*x*/*a*	*y*/*b*	*z*/*c*	Occ. factor	U_{iso}
Ba/Pb	2*d*	2/3	1/3	¼	0.56(2)/0.44	367(7)
Fe(1)	2*a*	0	0	0	1	98(8)
Fe(2)	2*b*	0	0	1/4	1	161(9)
Fe(3)	4*f*	2/3	1/3	0.5272(1)	1	89(6)
Fe(4)	4*f*	2/3	1/3	0.6899(1)	1	101(6)
Fe(5)	12*k*	0.1687(2)	2*x*	0.6082(1)	1	87(5)
O(1)	4*e*	0	0	0.6498(5)	1	83(22)
O(2)	4*f*	2/3	1/3	0.4455(5)	1	138(27)
O(3)	6*h*	0.1814(10)	2*x*	¼	1	171(23)
O(4)	12*k*	0.1571(7)	2*x*	0.0523(3)	1	81(14)
O(5)	12*k*	0.5014(8)	2*x*	0.1497(3)	1	102(13)

Table S5. Anisotropic thermal parameters (pm²) for $Ba_{0.56(2)}Pb_{0.44}Fe_{12}O_{19}$

Atom	U_{11}	U_{22}	U_{33}	U_{23}	U_{13}	U_{12}	U_{iso}
Ba/Pb	436(9)	U_{11}	219(9)	0	0	218(4)	363(7)
Fe(1)	89(11)	U_{11}	101(17)	0	0	45(5)	93(7)
Fe(2)	114(12)	U_{11}	240(20)	0	0	57(6)	156(9)
Fe(3)	82(8)	U_{11}	85(11)	0	0	41(4)	83(6)
Fe(4)	86(8)	U_{11}	115(12)	0	0	95(6)	95(6)
Fe(5)	73(6)	64(8)	109(7)	-1(1)	½ U_{23}	32(4)	83(5)

Table S6. Atomic coordinates, occupation factors and isotropic displacement parameters (pm²) for $Ba_{0.77(2)}Pb_{0.23}Fe_{12}O_{19}$

Atom	Site	*x*/*a*	*y*/*b*	*z*/*c*	Occ. factor	U_{iso}
Ba/Pb	2*d*	2/3	1/3	¼	0.77(2)/0.23	222(5)
Fe(1)	2*a*	0	0	0	1	63(6)
Fe(2)	2*b*	0	0	1/4	1	162(7)
Fe(3)	4*f*	2/3	1/3	0.5273(1)	1	67(5)
Fe(4)	4*f*	2/3	1/3	0.6900(1)	1	68(5)
Fe(5)	12*k*	0.1688(1)	2*x*	0.6082(1)	1	64(3)
O(1)	4*e*	0	0	0.6499(4)	1	69(19)
O(2)	4*f*	2/3	1/3	0.4454(4)	1	70(20)
O(3)	6*h*	0.1816(9)	2*x*	¼	1	124(18)
O(4)	12*k*	0.1567(6)	2*x*	0.0521(2)	1	64(11)
O(5)	12*k*	0.5025(7)	2*x*	0.1494(2)	1	71(11)

Table S7. Anisotropic thermal parameters (pm²) for $Ba_{0.77(2)}Pb_{0.23}Fe_{12}O_{19}$

Atom	U_{11}	U_{22}	U_{33}	U_{23}	U_{13}	U_{12}	U_{iso}
Ba/Pb	252(6)	U_{11}	161(7)	0	0	126(3)	222(5)
Fe(1)	63(9)	U_{11}	64(12)	0	0	31(4)	63(6)
Fe(2)	97(10)	U_{11}	294(18)	0	0	48(5)	162(7)
Fe(3)	59(6)	U_{11}	81(9)	0	0	29(3)	67(5)
Fe(4)	58(6)	U_{11}	88(9)	0	0	29(3)	68(5)
Fe(5)	44(6)	44(6)	89(6)	-4(5)	½ U_{23}	22(3)	64(3)
